# Supplementary material for: A Polish Version of the Boston Carpal Tunnel Questionnaire (BCTQ-PL) for Use Among Patients with Carpal Tunnel Syndrome Undergoing Physiotherapy: Translation, Cultural Adaptation, and Validation
Source: Healthcare (Basel). 2025 May 29;13(11):1288. doi: 10.3390/healthcare13111288 (PMC12154119; doi:10.3390/healthcare13111288)
Supplement: Supplementary file 1 [file healthcare-13-01288-s001.zip › healthcare-3622110-supplementary.pdf]

Table S1. Outcomes of the translation and cultural adaptation steps of the BCTQ to the Polish context

**Step 1. Preliminary translations**	
**BCTQ English version**	**Translations - T1 and T2**
Symptom Severity Scale	T1 Skala Dotkliwości Objawów<br>T2 Skala Oceny Objawów
SSS no. 4 Do you have numbness (loss of sensation) in your hand?	T1 Czy doświadcza Pan/Pani drętwienia dłoni (utraty czucia w dłoniach)?<br>T2 Czy doświadcza Pan/Pani drętwienie (brak czucia) w dłoni?
**Step 2. Translations' synthesis**	
**Translations T1 and T2**	**Translations' synthesis**
T1 Skala Dotkliwości Objawów<br>T2 Skala Oceny Objawów	T1-2 Skala Nasilenia Objawów
T1 Czy doświadcza Pan/Pani drętwienia dłoni (utraty czucia w dłoniach)?<br>T2 Czy doświadcza Pan/Pani drętwienie (brak czucia) w dłoni?	T1-2 Czy doświadcza Pan/Pani drętwienia (utraty czucia) w dłoni?
**Step 3 Back translation**	
**Translations' synthesis**	**Back translations (BT)**
FSS 4 Chwytanie słuchawki telefonu	BT1 Gripping a telephone receiver<br>BT2 Handling a phone<br>BT1-2 The necessity to adapt the item to technological progress was noticed and reported to the expert committee
FSS 8 Mycie i ubieranie się	BT1 Washing and dressing<br>BT 2 Showering and getting dressed<br>BT1-2 The need to adapt the item to ensure accurate semantic equivalence in the Polish language
**Step 4. Review by the committee of experts**	
**Original problematic item in the English version**	**Agreed item wording in the Polish version**
FSS 4 Gripping of a telephone handle	Chwytanie i używanie telefonu (Grabbing and using a mobile)
FSS 8 Bathing and dressing	FSS 8 Mycie się i ubieranie
**Step 5. Test of the pre-final version (patients with CTS)**	
**Item / response option in the pre-final Polish version**	**Item / response options in the final Polish version**
SSS 2 Jak często ból dłoni lub nadgarstka budził Pana/Panią w nocy w ciągu ostatnich dwóch tygodni?	SSS 2 Jak często ból dłoni lub nadgarstka budził Pana/Panią w ciągu typowej nocy podczas ostatnich dwóch tygodni?
SSS 10 Jak często drętwienie lub mrowienie dłoni budziło Pana/Panią w nocy w ciągu ostatnich dwóch tygodni?	SSS 10 Jak często drętwienie lub mrowienie dłoni budziło Pana/Panią w ciągu typowej nocy podczas ostatnich dwóch tygodni?
SSS – response option<br>Nie mogę wcale wykonać tej czynności z powodu objawów w dłoni lub nadgarstku	SSS – response option<br>Nie mogę wcale wykonać tej czynności z powodu objawów w bolesnej dłoni lub nadgarstku

Table S2. Boston Carpal Tunnel Questionnaire – Polish version (BCTQ-PL)

TABELA I
SKALA NASILENIA OBJAWÓW

Poniższe pytania odnoszą się do objawów występujących u Pana/Pani w typowym dwudziestoczterogodzinnym okresie w ciągu ostatnich dwóch tygodni (proszę zakreślić jedną odpowiedź na każde pytanie).

1. Jak dokuczliwy jest ból dłoni lub nadgarstka odczuwany przez Pana/Panią w nocy?
   1 Nie odczuwam bólu dłoni lub nadgarstka w nocy
   2 Łagodny ból
   3 Umiarkowany ból
   4 Silny ból
   5 Bardzo silny ból

2. Jak często ból dłoni lub nadgarstka budził Pana/Panią w ciągu typowej nocy podczas ostatnich dwóch tygodni?
   1 Nigdy
   2 Jeden raz
   3 Dwa lub trzy razy
   4 Cztery lub pięć razy
   5 Ponad pięć razy

3. Czy w ciągu dnia zazwyczaj odczuwa Pan/Pani ból dłoni lub nadgarstka?
   1 Nigdy nie odczuwam bólu w ciągu dnia
   2 Odczuwam łagodny ból w ciągu dnia
   3 Odczuwam umiarkowany ból w ciągu dnia
   4 Odczuwam silny ból w ciągu dnia
   5 Odczuwam bardzo silny ból w ciągu dnia

4. Jak często odczuwa Pan/Pani ból dłoni lub nadgarstka w ciągu dnia?
   1 Nigdy
   2 Raz lub dwa razy dziennie
   3 Trzy do pięciu razy dziennie
   4 Ponad pięć razy dziennie
   5 Ból jest stały

5. Jak długo średnio trwa epizod bólu występujący w ciągu dnia?
   1 Nigdy nie odczuwam bólu w ciągu dnia
   2 Krócej niż 10 minut
   3 Od 10 do 60 minut
   4 Dłużej niż 60 minut
   5 Ból jest stały przez cały dzień

6. Czy doświadcza Pan/Pani drętwienia (utraty czucia) w dłoni?
   1 Nie
   2 Odczuwam łagodne drętwienie
   3 Odczuwam umiarkowane drętwienie
   4 Odczuwam silne drętwienie
   5 Odczuwam bardzo silne drętwienie

7. Czy odczuwa Pan/Pani osłabienie w dłoni lub nadgarstku?
1 Brak osłabienia
2 Lekkie osłabienie
3 Umiarkowane osłabienie
4 Silne osłabienie
5 Bardzo silne osłabienie

8. Czy doświadcza Pan/Pani mrowienia w dłoni?
1 Brak mrowienia
2 Lekkie mrowienie
3 Umiarkowane mrowienie
4 Silne mrowienie
5 Bardzo silne mrowienie

9. Jak bardzo nasilone jest drętwienie (utrata czucia) lub mrowienie w nocy?
1 Nie odczuwam drętwienia ani mrowienia w nocy
2 Lekkie
3 Umiarkowane
4 Silne
5 Bardzo silne

10. Jak często drętwienie lub mrowienie dłoni budziło Pana/Panią w ciągu typowej nocy podczas ostatnich dwóch tygodni?
1 Nigdy
2 Jeden raz
3 Dwa lub trzy razy
4 Cztery lub pięć razy
5 Ponad pięć razy

11. Czy ma Pan/Pani trudności w chwytaniu i używaniu małych przedmiotów, takich jak klucze lub długopisy?
1 Brak trudności
2 Niewielka trudność
3 Umiarkowana trudność
4 Duża trudność
5 Bardzo duża trudność

TABELA II
SKALA STANU FUNKCJONALNEGO

Czy podczas ostatnich dwóch tygodni, w ciągu typowego dnia objawy w dłoni i nadgarstku powodowały u Pana/Pani trudności w wykonywaniu poniższych czynności? Proszę zakreślić cyfrę najlepiej odpowiadającą Pana/Pani możliwości wykonania danej czynności.

Czynność	Brak trudności	Niewielka trudność	Umiarkowana trudność	Duża trudność	Nie mogę wcale wykonać tej czynności z powodu objawów w bolesnej dłoni lub nadgarstku
Pisanie	1	2	3	4	5

Zapinanie guzików w ubraniach	1	2	3	4	5
Trzymanie książki podczas czytania	1	2	3	4	5
Chwytanie i używanie telefonu	1	2	3	4	5
Otwieranie słoików	1	2	3	4	5
Prace domowe	1	2	3	4	5
Noszenie toreb z zakupami	1	2	3	4	5
Mycie się i ubieranie	1	2	3	4	5
